# Patients’ and Members of the Public’s Wishes Regarding Transparency in the Context of Secondary Use of Health Data: Scoping Review

**DOI:** 10.2196/45002

**Published:** 2023-04-13

**Authors:** Annabelle Cumyn, Jean-Frédéric Ménard, Adrien Barton, Roxanne Dault, Frédérique Lévesque, Jean-François Ethier

**Affiliations:** 1 Département de médecine Faculté de médecine et des sciences de la santé Université de Sherbrooke Sherbrooke, QC Canada; 2 Groupe de recherche interdisciplinaire en informatique de la santé Faculté des sciences/Faculté de médecine et des sciences de la santé Université de Sherbrooke Sherbrooke, QC Canada; 3 Faculté de droit Université de Sherbrooke Sherbrooke, QC Canada; 4 Institut de recherche en informatique de Toulouse Toulouse France

**Keywords:** transparency, information, means of communication, public, patients, secondary use, health data, learning health systems

## Abstract

**Background:**

Secondary use of health data has reached unequaled potential to improve health systems governance, knowledge, and clinical care. Transparency regarding this secondary use is frequently cited as necessary to address deficits in trust and conditional support and to increase patient awareness.

**Objective:**

We aimed to review the current published literature to identify different stakeholders’ perspectives and recommendations on what information patients and members of the public want to learn about the secondary use of health data for research purposes and how and in which situations.

**Methods:**

Using PRISMA-ScR (Preferred Reporting Items for Systematic Reviews and Meta-Analyses extension for Scoping Reviews) guidelines, we conducted a scoping review using Medline, CINAHL, PsycINFO, Scopus, Cochrane Library, and PubMed databases to locate a broad range of studies published in English or French until November 2022. We included articles reporting a stakeholder’s perspective or recommendations of what information patients and members of the public want to learn about the secondary use of health data for research purposes and how or in which situations. Data were collected and analyzed with an iterative thematic approach using NVivo.

**Results:**

Overall, 178 articles were included in this scoping review. The type of information can be divided into generic and specific content. Generic content includes information on governance and regulatory frameworks, technical aspects, and scientific aims. Specific content includes updates on the use of one’s data, return of results from individual tests, information on global results, information on data sharing, and how to access one’s data. Recommendations on how to communicate the information focused on frequency, use of various supports, formats, and wording. Methods for communication generally favored broad approaches such as nationwide publicity campaigns, mainstream and social media for generic content, and mixed approaches for specific content including websites, patient portals, and face-to-face encounters. Content should be tailored to the individual as much as possible with regard to length, avoidance of technical terms, cultural competence, and level of detail. Finally, the review outlined 4 major situations where communication was deemed necessary: before a new use of data, when new test results became available, when global research results were released, and in the advent of a breach in confidentiality.

**Conclusions:**

This review highlights how different types of information and approaches to communication efforts may serve as the basis for achieving greater transparency. Governing bodies could use the results: to elaborate or evaluate strategies to educate on the potential benefits; to provide some knowledge and control over data use as a form of reciprocity; and as a condition to engage citizens and build and maintain trust. Future work is needed to assess which strategies achieve the greatest outreach while striking a balance between meeting information needs and use of resources.

## Introduction

### Potential Benefits From the Secondary Use of Health Data

In recent decades, the potential benefits of secondary use of health data have been highlighted in many different contexts, including health systems governance, quality improvement [1], point-of-care decision-making [2-4], and research [5]. While a unique, unanimously accepted definition of secondary use remains elusive, we will operate under the premise that it covers any use beyond the initial intent of data collection. Given the resources required to generate health data, using them to their fullest extent to improve health is a laudable goal. Nevertheless, health data often describe an individual, and thus their autonomy regarding the use of data also needs to be considered [6]. For individuals to be able to weigh in on the use of data about them, they at least need to be aware that data generated for a primary reason (eg, during a consultation with a physician) have values above and beyond the specific context for which they were created and can therefore be used for secondary goals such as research projects or systems policy research. As mentioned previously, the secondary use of health data covers a broad landscape, and the scale of expected benefits (eg, How much? How soon? Who?) varies from one use to the other, which could modulate how individuals react to different types of secondary use.

Therefore, while the potential of secondary use of health data for research purposes is seen as promising to many stakeholders in the academic and health care worlds, trust regarding data sharing in novel contexts should not be considered as a given [7]. Indeed, despite substantial evidence that most individuals support the secondary use of health data, this support is neither unanimous nor unconditional [6,8-11]. Stakeholders including patients and members of the public express different needs that touch upon trust and agency [12]. Controversial initiatives such as care data in England [13], the National Electronic Health Record System in Australia [14], and other incidents involving unconsented and unauthorized use of data [15-17] have contributed to some mistrust in the population, which may then tend to develop greater sensitivity to the risks associated with data sharing and health data in general [7,12]. In addition, few people seem to be aware that the secondary use of their health data is already permissible in certain circumstances [18]. In all these situations, some stakeholders perceived a lack of transparency. Although transparency is unquestionably identified as an essential element of social license regarding the secondary use of health data, it is not always clearly defined. For example, Kisekka et al [19] showed that information accuracy and availability are important parameters for the adoption of e-portals; however, an open question is which kind of information is expected by the stakeholders and under which modalities of communication. Furthermore, technological advances offer new ways for transparency to become tangible and bring forth strategies for more open communication [20]. Therefore, there is a real need to better understand the actual expectations regarding transparency with regard to secondary use of health data.

### Potential of Learning Health Systems

The fact that a research project is targeting a common health issue such as heart attacks does not guarantee a rapid and substantial benefit as an important justification for valuing the secondary use of health data. For example, in 1982, studies identified a class of medication, namely β-blockers, as useful in reducing mortality in the context of heart attacks. Nevertheless, it took more than 20 years to confirm the widespread adoption of this simple and relatively inexpensive intervention [21].

To increase the pertinence of projects undertaken, the rapidity at which the resulting new knowledge is put into action, and therefore the benefits to patients, learning health systems (LHSs) have been proposed. They are a model that embodies how linking data from clinical care and research can create new knowledge and improve clinical decision-making [22]. Recent publications have reported the success of early LHSs [1,23,24], which is encouraging. Moreover, this integration of care delivery, research, and effective knowledge transfer can be implemented as described in the study by Faden et al in 2013 [25], in which a new ethical contract is based on a large engagement of all stakeholders (including patients), transparency (both on ongoing activities and data supporting them), and accountability to demonstrate benefits for the participants. While communicating about secondary data use is important, it is particularly essential in an LHS context.

### Aims

This scoping review aims to describe how transparency is portrayed by various stakeholders regarding the secondary use of health data in a research context. Specifically, we wanted to address the following questions: (1) What information do patients and members of the public want to learn about the secondary use of their health data for research purposes (what)? (2) How do patients and members of the public want to interact with this information (how)? and (3) In which situations (when)? The objectives of the scoping review were elaborated by a team composed of an expert in health informatics (J-FE), an expert in research ethics (AC), a legal expert (J-FM), and an expert in ethics and philosophy of science (AB).

The study aims to capture a broad landscape of the literature to serve as a cornerstone of the upcoming consultations with patients and members of the public to identify the operational requirements of a transparency portal as LHSs are deployed in the province of Quebec. Therefore, resources specifically targeting an LHS context were identified when available, but the search was not restricted to only these as the LHS field is relatively young.

## Methods

### Overview

We addressed our research objectives by performing a scoping review following the 6-stage methodology framework by Levac et al [26] (based on the framework initially proposed by Arksey and O’Malley [27]), which we reported in accordance with the PRISMA-ScR (Preferred Reporting Items for Systematic Reviews and Meta-Analyses extension for Scoping Reviews) framework [28] (the PRISMA-ScR checklist is available in Multimedia Appendix 1). Our aim was to obtain a large spectrum of articles, from original research to opinion papers, law articles, and workshop reports. Our review protocol was finalized in July 2021, but was not made publicly available.

### Definition of Health Data in This Review

We defined health data as data generated either in the context of health care, whether found in patient charts or connected objects, or in the context of health care research to capture perceptions about all kinds of secondary uses. In these secondary uses, we included approved future uses of biobank data because the literature on that matter was quite relevant to our research question.

### The Concept of Transparency in This Review

The term “transparency” characterizes a process of information communication that follows certain norms about the amount, type, and framing of information that is shared. For example, the process of informing a person about access to one’s health data by third parties through a poster in a health professional’s office might be characterized as aiming at being transparent. By extension, the term “principle of transparency” can refer to a normative principle that instructs to communicate information in a transparent way (in the first sense). Thus, the 2 senses of the term “transparency” are closely linked. Since the 2 core notions of transparency are its relevance and its easiness to understand, this suggests that an investigation of transparency should concentrate on questions such as “What information should be conveyed?” “How should it be conveyed?” “In which circumstances should it be conveyed?”

### Search Strategy

An extensive literature search was performed for articles up to November 2022 in the following bibliographic databases: Medline, CINAHL, PsycINFO, Scopus, Cochrane Library, and PubMed. The search strategy was developed with the assistance of a university librarian and was adapted to each database (the detailed search strategies are provided in Multimedia Appendix 2). The 5 core concepts used in the search strategy were patient, public, citizen (and synonyms); health data, medical data, data use, data sharing (and synonyms); attitude, view, perspective, opinion, position (and synonyms); information, transparency, communication, dissemination, awareness, notification, education (and synonyms); and research, secondary use, LHS (and synonyms). We did not have to limit the time span.

### Eligibility Criteria and Screening

Two reviewers (RD and FL) independently screened the titles and abstracts of all articles identified by the search strategy (after removing duplicates), followed by a full-text review of the remaining articles. To perform an in-depth and broad research, no article was excluded based on year, type, or location of publication. The eligibility criteria are presented in Textbox 1.

**Eligibility criteria.**

Inclusion criteria

- Language and article availability
  French
  English
  Full-text availability
- Article type
  Original articles
  Commentary or opinion papers
  Policy or law articles
  Ethics articles
- Population
  Reporting at least patients, members of the public or other experts’ (eg, health care professionals and researchers in various domains) point of views or recommendations
- Outcomes
  Reporting at least 1 of the following elements: What information to communicate to patients or members of the public; how to communicate the information (eg, format and support).
- Context
  Secondary use of data
  Uses in research
- Data type
  Health data such as hospital data, electronic record data, administrative data, or medical data

Exclusion criteria

- Language and article availability
  Other language
  Abstract only
- Article type
  Review articles (Review articles that met the other inclusion criteria were screened to include potential studies of interest.)
  Study protocols
- Context
  Primary use of data (eg, for health follow-up)
  Other secondary uses of data (eg, quality improvement, safety monitoring, clinical decision-making, or policy making)

The 2 reviewers first performed a joint screening on a 10% sample of the articles to familiarize themselves with the selection criteria and to standardize their approach. Every discrepancy between the 2 reviewers was discussed to obtain a consensus. If a consensus could not be reached, a third reviewer (AC or J-FM) was consulted. The team met weekly to discuss the progress of the literature review. The search and screening process was initially completed in July 2021 but was updated up to November 2022 following the same methodology to capture and include recent publications.

### Data Extraction and Analysis

The extraction, organization, and analysis of information from the articles included in this scoping review was performed using NVivo 11 Pro software (QSR International), a tool to conduct extensive literature reviews [29]. On the basis of the aforementioned research questions and objectives, the research team developed a codebook with an initial tree of nodes to chart the data into NVivo. This codebook also includes the coding procedure and definitions of the nodes for the reviewers responsible for the data extraction. All selected articles were imported into NVivo software. Four reviewers (RD, FL, AC, and 1 research assistant) coded all the articles, a process that was subsequently validated by 2 reviewers (AC and J-FM). All reviewers received detailed training in the software, project, and codebook. They all simultaneously coded a sample of the articles to familiarize themselves with the tree of nodes and coding method. During training, the reviewers’ coding was compared with determine whether their approach to data extraction was consistent, and discrepancies were discussed in team meetings. This process was repeated until all reviewers were comfortable with the coding. The coding was performed in an iterative manner to allow the emergence of new concepts and themes in the tree of nodes. Weekly team meetings were held between reviewers to discuss and validate coding decisions as well as emerging themes. The information was subsequently analyzed to examine what information, time frame, and means of communication were proposed, recommended, or desired for various research activities regarding the access and secondary use of health data for research purposes. We also aimed to identify topics that were not addressed in the literature for future work. The following 3 themes emerged from a text analysis: content to communicate, how to communicate, and situations in which communication becomes relevant. A fourth theme also emerged, namely, the impacts of communication, which were further divided into potentially negative, neutral, or positive impacts. This last theme was not directly linked to our research question and was the focus of a separate article. Note that the literature update from July 2021 to November 2022 did not bring any new concepts to light but rather confirmed that we had reached theoretical data saturation.

## Results

### Search Outcome and Bibliographic Overview

**Figure 1:**
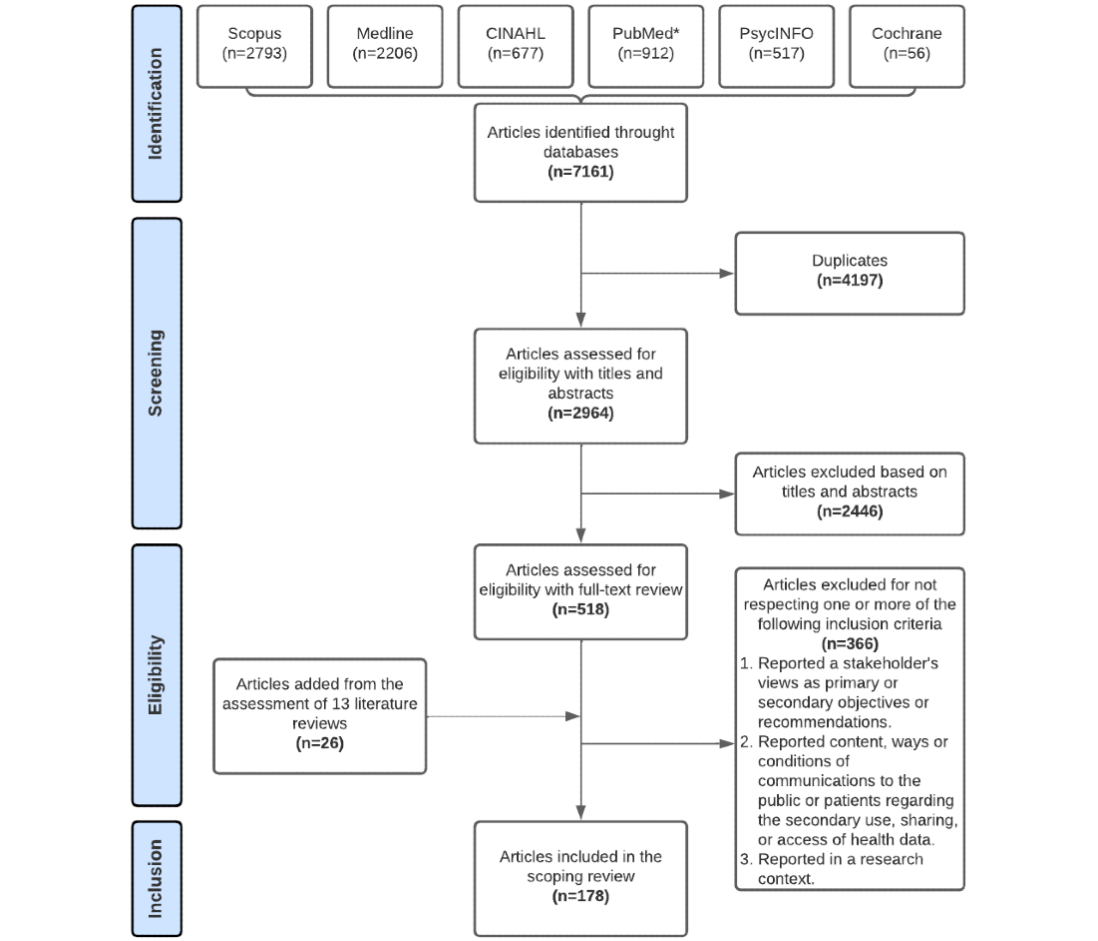
Article selection process. *Restricted to publications in the last 2 years for more recent indexation.

The detailed selection process is illustrated in Figure 1. After removing duplicates, 2964 articles were identified through the database search. The screening of titles and abstracts led to the assessment of 518 articles in a full-text review. In total, 152 original articles and 13 review articles met our inclusion criteria. The manual assessment of the references from the review articles added 26 original articles. Only original articles of those reviews that met our inclusion criteria were included in the final selection of this scoping review. Finally, 178 articles were included in this scoping review.

The main characteristics of the articles included in this review are presented in Table 1, and 178 complete references are presented in Multimedia Appendix 3 [6,8,11,18,19,30-202].

**Table 1:** Characteristics of articles included in the scoping review (n=178).

Characteristics			Articles, n (%)
**Year of publication**			
	2002-2006	9 (5.1)	
	2007-2017	71 (39.9)	
	2018	10 (5.6)	
	2019	27 (15.2)	
	2020	22 (12.4)	
	2021	24 (13.5)	
	2022	15 (8.4)	
**Continent where study was conducted^a^**			
	North America	86 (48.3)	
	Europe	69 (38.8)	
	Asia	6 (3.4)	
	Oceania	5 (2.8)	
	Africa	4 (2.2)	
	Worldwide	8 (4.5)	
**Study design^b^**			
	Qualitative	91 (51.1)	
	Quantitative	52 (29.2)	
	Mixed approach	9 (5.1)	
	Commentary	9 (5.1)	
	Law and politics	16 (8.9)	
	Descriptive	1 (0.6)	
**Perspectives reported in the study^c^**			
	Public	68 (38.2)	
	Patients	59 (33.1)	
	Researchers	26 (14.6)	
	Law and politics	22 (12.4)	
	Ethics	15 (8.4)	
	Health care	11 (6.2)	
	eHealth or informatics	8 (4.5)	

^a^North America: Canada, United States; Europe: United Kingdom, Iceland, Ireland, Sweden, Italy, Germany, Denmark, Switzerland, Norway, Portugal, the Netherlands, Belgium, France, and Finland. Asia: Saudi Arabia, Taiwan, China, India, and Japan. Oceania: New Zealand and Australia. Africa: Ghana, Uganda, Zambia, Kenya, and Singapore. Worldwide: studies with multiple countries involved.

^b^Qualitative: workshops, interviews, focus groups, councils, and panels; Quantitative: survey and Delphi; Mixed approach: combination of quantitative and qualitative designs; Commentary; Law and politics: ethical, legal, or governance frameworks, guidelines, requirements, and other policies; and descriptive study.

^c^Not mutually exclusive. Public: Indigenous communities, minority community members, older adults, blind and low-vision communities, and early adopters of emerging technologies. Patients: susceptible, rare diseases, cancer, cardiac, Parkinson disease, mental health, pediatric (parents and families), and representatives of patients’ organizations. Researchers: research participants, students, researchers, recruiters, sponsors, investigators, scientific staff, data infrastructure employers and managers, and research governance experts. Law and politics: policy makers, legal professionals, and regulatory staff. Ethics: ethicists and research ethics committee members. Health care: health care professionals, health managers, and health systems leaders. eHealth or informatics: eHealth experts, device or app developers, and data-sharing experts.

### What to Communicate?

There were 2 distinct types of content that emerged from this review. We refer to them as “generic content” (communication on broader aspects than the secondary use of their own personal health data) and “specific content” (communication on the secondary use of their own personal health data).

#### Generic Content

##### Overview

Table 2 lists an overview of the types of generic content that various stakeholders wish to see communicated with exemplary citations extracted from the studies reviewed.

**Table 2:** Types of generic content: description and examples of stakeholders’ perspectives.

Type of generic content	Description	Exemplary citations of different stakeholders extracted from reviewed studies
General information on secondary use of health data [11,19,30-61]	Education around data types and secondary uses [49,54-56,59-61] Education on Research Ethics Board role [11,46,50-53]	“[...] I think that education in general is a really good tool for the layperson to understand what’s happening and I just think that that’s probably the key, educating away the fears but also disclosing—what are we responsible for? What could happen? It’s going to help people trust what you’re doing a little more too” (biobank participant [39]). “Participants clearly identified a number of areas where there was a need for more knowledge and work around data sharing. [...] There were four main areas where greater knowledge was required: (1) clarity regarding the legal and clinical implications of shared data for patients and providers, (2) an understanding of legislation across Canada, (3) decision-making about what data are needed and (4) being aware of the context of electronic medical records [EMR] data” (Terry et al [35] on the views of a variety of stakeholders).
Information on data governance [11,31,33,34,36, 40-42,49,52-54,57, 59,60,62-118]	Who: identity of data overseers [11,31,41,49,53,76,83,85,98, 100-102,115] and data users [11,31,52,73-77,79-81,86,91,113,114, 117,118] What and what for: type of data used and type of secondary use [53,54,59,60,62,75,86,94,108-110, 117,118] With whom: data-sharing practices [59,60,65,90,97,105,106,111,118] How: sources of funding [95,96], safeguards [34,53,73,103,104,112]	“Respondents identified biobank objectives, governance structure and accountability as the most important information to provide participants. Respondents’ concerns about biobanking generally centered around the control and ownership of biological samples and data, especially with respect to potential misuse by insurers, the government and other third parties” (Joly et al [64] on the views of members of the public). “I’m just trying to say there is this framework, you know we say that there is a governance system in place which will protect the patient and we can look at them like we do the ﬁnancial institutions and we’re quite happy with how they exist, well they’re quite well developed. There’s a framework around this and we want some assurance” (patient with a rare disease [67]). “Well, I did not know how freely they could share the information, that they are actually sharing them with payers. So, something needs to be done with that because we have a right to know where our information is going [...]” (oncology patient [114]). “I guess, for me I think it’s not so much the party accessing the data, but rather how is the data being used for and for what purpose. So knowing that, then I’m able to make a better decision as in whether I want to participate. ... if it’s from a big pharmacy company, then I think it may be for a commercial gain, but again it still help people. So I guess it’s still the purpose, how the data being used, the purpose what is it used for” (member of the public [94]). “Similar to other findings [...], our discussants emphasized, however, that disclosure of data-sharing practices was important in order to make a truly informed decision and fulfill the fundamental ethical principles of participant autonomy and respect” (Haga and O’Daniel [63] on the views of members of the public). “Overall, workshop participants felt that if they knew more about the processes and safeguards in place, they might feel more empowered, and hence more open and trusting in the decision-making process around data collection and sharing (and may not, therefore, need to opt-in)” (Ipsos [112] on the views of members of the public).
Information on ethical and legal aspects [31,33, 35,39,40,52,53, 60,63,64,71,73,75, 77,80,81,85,86, 103,111,118-131]	Legal framework [35,81,124,126,127] Confidentiality and privacy measures [33,60,63,71, 75,80,86,117,118,123,129,130] Security breach [31,52,60,103] Penalties for misuse [31,39,60,73,77,85,103,111,124,128] Participant rights [73,120-122,131] Intellectual property rights [52]	“It was also suggested that there would be some benefit in raising public awareness of the complex legal environment surrounding data sharing and that this might demonstrate the legitimacy of researchers’ access to data” (Mamo et al [40] on the views of members of the public). “How it is being used, how I am protected from corrupt or evil activities, and what precautions are taken to protect it” (member of the public [64]). “To protect our privacy in a world where we no longer control our data, we must obfuscate health data, penalize the misuse of health data, and improve transparency around who shares our data and for what purposes” (expert opinion [111]).
Information on technical aspects [33,34,38, 40,52,53,62,63,73, 78-80,82,83,86, 91-93,95-97,103,106, 118,120,130,132,133]	Data linkage [53,83,96,103,132] Types of data sets [34,62] Whether medical records are accessed [78] How data are shared [38,80,91,92,106,130] Data security [52,73,79,97,103,117,120,133] Duration of data storage and how they are stored [78,93,95,97,118]	“I would want to know what kind of security the central network is using. Are they using any type of encryption at all, who has access to the system? How do they maintain that type of access, you know, just general [questions]” (patient [40]). “Patients also brought up the importance of restricting data access, oversight of such restrictions and voiced specific questions about data security, for instance, wanting details on how the data would be transferred. Some patients expressed uncertainty about current practices; as one patient said, ‘I don’t know who has access to my information’” (Mazor et al [106] on the views of patients). “Kept in a very safe location. I hate qualifiers like that. It doesn’t make me feel very safe. I: What would make you feel safe? P: When I see ‘will be kept in a very safe location.’ I would want specifics” (patient [73]).
Information on scientific aspects [11,31,46, 53,64,65,68,73, 75-77,79,85,89, 92,93,95-97,99, 115,132,134-136]	Nature of the research and objectives [11,31,64,68,76,77,79,95, 97,115,132,136] Research methodology [79,93] Risks and benefits [46,53,65,73, 75,77,79,85,89,92,95,96,99,134] Data validity and possible biases [75,79,96]	“Around half of the respondents want to receive an easily understandable summary of project (51%) and information about the management rules (49%)” (Courbier et al [76] on the views of patients with rare diseases). “It would be very helpful to the reader and potential study subject to have some, at least, some examples of the type of research the researchers intend to do” (patient [73]). “[My mobile data] shows a terrible step count, but that’s because I don’t hold my phone while playing netball, long walks etc” (member of the public [75]).

##### General Information on Secondary Use of Health Data

The need for general knowledge about information systems and data-driven research was a transversal theme in our review (Table 2, row 1), whether in the context of biobanking [19,30-45,71,83,84], precision medicine [92], mining of health data [34,44,49,72,115], or data sharing with the industry [71,73,75,134]. Some stated that the need for education extended to all stakeholders, including patients, research participants, and researchers. Clinicians were also targeted for education on how to integrate genomics into clinical care [40,55,137] and training in information technologies [120]. Members of the public and research participants highlighted the value of [31,83]

communicating positive messages about how data are used: ‘promoting the success stories’, And I can see an advantage in updates because I think it creates a positive view of things, a positive view while there’s so much bad information. You know that here’s a group of people working for the human good and you’ve participated in it, you know. It’s uplifting really

Biobank participant

##### Information on Data Governance

The second set of generic content is related to data governance (Table 2, row 2). Comprehensive and transparent governance emerged as a key point in the literature from biobanking [64,69,78,102] and appeared to apply to more novel uses of data; for example, secondary use of anonymized mobile phone data [33,65,66,80], unregulated mobile apps [81], novel data linkages [75,132], sharing genomic data [57,85,91], distributed data networks [40,109,138], or artificial intelligence [79,108,139]. The most common content elements pertain to the structure of governance, nature of secondary use [42,76], and the identity of data users, in particular if sharing with the industry [38,40,61,75,93,97,104]. In a recent survey, knowing the names of the individuals in charge of data governance was particularly important if identifiable health data were being accessed in the context of data mining [115]. In the same line of thought, an analysis on 836 respondents by Kisekka et al [19] concluded that in the context of e-portals and health technology use, “[...] individuals worry more about who possesses the right to access their health data (i.e., who, what, when, and why) than the mechanisms used to safeguard data from unauthorized access.” Of interest, the composition of the governance body; distribution of power; presence of a data overseer (at times referred to as an ombudsman); and the integration of patients, citizens, or the community in governance were suggested as important information to foster trust.

##### Information on Ethical and Legal Aspects

Stakeholders wished to obtain some information on the legal frameworks protecting the data, confidentiality and privacy measures, penalties for misuses, participant rights, and intellectual property rights (Table 2, row 3). The participant rights most mentioned were related to a need for control over future use, with a focus on individual consent. In a survey of public perspectives on genomic data sharing with responses from 36,268 individuals across 22 low-, middle-, and high-income countries, the right to withdraw one’s data was ranked highly as a measure to increase trust (second to information on who would benefit from the data sharing) [85].

##### Information on Technical Aspects

Many raised the issue of technical information, such as measures taken to protect the data (Table 2, row 4). Fewer stakeholders wished for information about data linkage, how data are shared, and information security.

##### Information on Scientific Aspects

Some members of the public and patients suggested that information regarding the nature of the research would be welcome with a focus on general objectives [64] and benefits for the self or society [33,37,85,99,134] (Table 2, row 5). Stakeholders occasionally raised the issue of communicating measures taken to ensure data accuracy or validity [75,79,96].

#### Specific Content

##### Overview

Table 3 presents the major types of specific content that emerged from our review. Of note, when the comments concerned specific content, they emphasized the motivation for receiving such content. This was different from the comments on generic content that focused more on details around the type of content; hence, the difference in headers for the second column of Tables 2 and 3.

By far, the most frequent elements cited in the literature for specific information were related to the return of results from individual tests (Table 3, row 2) and the sharing of one’s data (Table 3, row 4).

**Table 3:** Types of specific content: motivations and examples of stakeholders’ perspectives.

Type of specific content	Motivation for receiving this content	Exemplary citations of different stakeholders extracted from reviewed studies
Information updates on the use of one’s data [48,51,68,83,95, 105,110,131,138, 140-145]	To remain informed [68,110,131,138,142] To remain engaged [68,78,87,129,140,144] As a form of reciprocity [83,117,145]	“I’d probably just want to be told that the study had expanded a little bit... that it was something different. Yeah, to keep everything above board. I would still say go ahead and use it, but... provided that the patient is aware” (patient [68]). “Concerning genetic data, all interviewees thought that next-of-kin should be informed about the fact that post-mortem genetic data analysis is taking place and be given the choice to be contacted about findings with potential relevance for their own health, if no prior preferences had been reported by the deceased” (Bak et al [54] on the views of patients and family members). “Well, it is all about giving and taking. You are giving information about yourself, about your state of health, in the end intimate details. And in return I want something back [...]” (member of the public [83]).
Information on results from individual tests [41, 51,54,64,69,70, 78,80,83,98, 118,119,127,140, 144,146-154]	For follow-up on one’s health [51,54,70,98,127,144,146,147] As a form of reciprocity [41,78,83,118,146] As a condition for participation in research [80,83,148] To mitigate concerns about privacy [119]	“I never heard any results. Our specimens [are] just being kept, being used however they might. What I would like to see is if specific tests are run. I would like to know the results” (research participant from an Indigenous community [144]). “I thought it would be great if I could delve into the relationship between my... genealogy and my cancer” (patient [146]). “To participate in a study where you get specific results would be very, for me, very positive. It would make me feel that I am contributing more instead of being lumped into this mass of people” (patient [146]).
Information on global research results of projects that used one’s data [41,47, 49,50,53,54,69, 76-78,80,81,92, 97,105,118,127, 128,135,138, 143,144,146, 155-160]	For transparency and to increase trust [36,49,53,77,78,89,97, 105,118,128,143,144] To help individuals or communities make informed choices about their health [47,50,78,92,127,129] As a gesture in return for participation [51,53,54,76,81,118,143,157] To share successes and increase research efficiency [41,77,83,105,138,143] If individual results are not available [146]	“[...] I think if I was to take part in anything like this I’d like to be able to see how the research was actually being used and its effects within society and how it’s helping people; that would be quite important for me to get something back” (biobank participant [83]). “When asked directly if they would like to be informed about the outcome of a data-sharing project in which they are participating, almost 100% of the respondents (99.7%) answer positively” (Courbier et al [76] on the views of patients with a rare disease). “Yes, okay, you’re going to share your data, but now we want you to share the results, positive or negative... One of the conditions for say getting our data is you have to share it and that shareable thing can be shareable with the public as well” (Parent of participant in a pediatric repository [138]). “It makes you feel like... what you’ve done is helpful and meaningful” (biobank participant [157]). “I’d like to know the results and whatever the issue is, how we can help communities, how we can help one another. Just what the next steps are. Where do we go from here?” (research participant from an Indigenous community [144]). “I mean, I would prefer linked because obviously there’s personal interest there, but if it’s done without that then I’d still be interested in the overall results” (patient with cancer [146]).
Information on the sharing of one’s data [11,45, 48,51,60,62,63, 67,73-75,78, 80,81,83,86, 88,95,99,100, 105,113,115, 118,119,121, 135,140,141, 143,155,158, 159,161-166]	To retain control over future use [45,67,73,75,81,83,88,99,100,113,118, 119,121,143,155,159,165] To know who can access or has accessed the data [63,74,80,161] To enact ongoing consent [11,48,60,95,105,140,141,143]	“I don’t need to manage it but do want to know who and when they check my file. That way I can decide whether grant access or not” (patient [45]). “Knowing what they’re doing or what they’re planning to do. To know exactly what everything [is that] they’re doing… and when and how it’s been used. Because, like I said, because it’s her genes, her stuff—you know” (parent [155]). “One participant said they would need to know, ‘exactly who, where, [and] how my information will be used [...]’” (Franklin et al [80] on the views of a patient with cancer). “I’d like to be notified anytime anybody accesses my medical records. Even if it’s my primary care physician... I’d either be notified through email or whenever you log on... When you log on, you should be able to see a list of everybody who’s accessed your file. If it’s electronic, you’d be notified if they’re trying to access something that’s more confidential” (patient [74]). “I think however they plan to [share the data]—they should inform so that you know what they are doing, and [where] it’s going to go—any method that they use” (member of the public [63]). “Yeah well, I feel if it’s confidential it’s confidential... and it’s anonymous, so… I suppose maybe I’d prefer to know personally... but then if you never know it is going to be released then it’s not going to bother you. But personally, I would prefer to know” (patient [161]). “I would like to be asked because if I think it’s important and it can help some sick people to be healed, yes, there’s no problem. But if I see that it’s not relevant and that it could be a bit of anything, then I might refuse” (member of the public [11]).
Information on how to access one’s data [44,53, 60,74,79,85, 99,100,109, 111,129,167, 168]	To be able to analyze one’s data [74,111,129,167] To be able to verify one’s data [44,53,60,79,85,109] To be able to withdraw data [85,168]	“If they collect the data, you should have some sort of report. You could say when something is missing” (member of the public [79]).

##### Information Updates on the Use of One’s Data

Patients and members of the public proposed that information updates be sent at the time of secondary use of their data (Table 3, row 1). Some referred to a need for control over future use, whereas others saw the ongoing relationship as a path to engagement or as a form of reciprocity. Of note, the need for information and control appeared prominently in certain groups, including racial and ethnic minorities [18,38], people who have lived through experiences of exclusion [169], and members of Indigenous communities [78,92,144].

##### Information on Results From Individual Tests

Across the literature, patients and members of the public alike expressed a strong expectation for being informed of results from tests conducted in research contexts (Table 3, row 2), even when it only concerned a marker for disease susceptibility [56], an indication of increased or decreased risk [153], exploratory (for example microbiome) research [127], and even when not performed in a certified laboratory [148]. This expectation was often merged with the request of the right to access one’s clinical data, which is outside the scope of the secondary use of health data for research. Clinicians and researchers, perhaps more cognizant of the distinctions between results that are part of a diagnostic process and nonvalidated research results, were less unanimously in support of the return of results [127,156,170].

In a cross-sectional study of the general Dutch population, participants wanted to receive both individual and aggregated results, with a preference for the former [69]. Notwithstanding this important preference for the return of individual results, many underlined the associated challenges, including the interpretation of results by the individual participants [127,146,152], and to a lesser extent, the risk of breach of confidentiality [150]. In addition, other factors seemed to influence preferences regarding the return of individual results, such as whether the medical condition was rare [76]; whether a health professional would be available to explain the results [69]; whether it had been agreed upon at the time of initial consent [81,91,163] (eg, at time of broad consent in the context of a biobank); whether there would be sharing of data with third parties [97], in particular those belonging to nonregulated, nonresearch settings [81]; and whether there might be logistical hurdles or excessive use of resources [146,152,170]. Indeed, many biobank participants may be primarily driven by altruistic motives and do not wish to allocate excessive resources to the return of results [83]. A participant in the study by Richards et al [157] stated the following:

Because the most important thing is to find, um, is the research itself. That’s the most important thing. So, to me, getting updates on what’s going on is a nice to have, but it’s not a must have.

Biobank participant

On the other hand, the return of results seemed to mitigate privacy concerns for other biobank participants [119].

##### Information on Global Research Results

The return of global research results of projects that used one’s health data (Table 3, row 3) was another important theme for several stakeholders, including members of Indigenous communities. Return of global results, whether positive or negative [138], feeds interest, fosters trust, and can permit individuals and communities to take more informed decisions. Participants from Indigenous communities underlined how providing information would be the way to gain and maintain their communities’ trust in research [78,89,144]. It was also seen as a gesture of gratitude in return for participation. Interestingly, in a survey of 80 Dutch researchers involved in biobank research, 23% disagreed in part or completely with the statement: “Participants have to be informed about aggregate research results” [156].

This opinion was echoed by 81.6% of patients with cardiovascular disease who responded to a survey distributed by the European Heart Network [135].

##### Information on How to Access One’s Data

Information on how to access one’s data (Table 3, row 5) was not frequently mentioned in the context of governance but seemed to be increasingly mentioned in recent years by members of the public in relation to a need to take control of one’s health, ensure data accuracy, and exert some control in the context of novel health data uses [44,79,85] or sharing of genomic data [155]. In the context of data mining, Watson and Payne [44] stated the following:

Structures to allow individual access are required to address inaccuracies in the data and to provide a sense of fairness and comfort in knowing that there is some recourse to address problems.

### How to Communicate?

#### Overview

Stakeholders proposed several characteristics of communications to ensure that they were effective (Table 4).

**Table 4:** Characteristics of communications: description and examples of stakeholders’ perspectives.

Characteristic of communication	Description	Exemplary citations of different stakeholders extracted from reviewed studies
Frequency [11, 18,33,36,38,49, 59,74,76,78,79, 81,100,105,125, 140,156,171-173]	Periodically, on an ongoing basis [33,36,38,74,76,78,79,81,140,156, 171,172] Linked to health care encounters [18,49,59,100,125] Upon request [78,105]	“The most appropriate approach would be to design consents and notices that are like that as well—real-time, updated, frequently communicating with you and letting you know not only how your data is going to be used and how it will be protected privacy and security wise... I think a consent information type notice should happen regularly [and] keep you engaged in understanding the continued use of this data” (regulatory expert opinion regarding unregulated mobile app research [81]). “At least once a year. If nothing else, you know what is going on” (patient from a US Veterans Affairs facility [77]). “Maybe every half a year, or maybe even once a month [...]. It would be good every six months to get follow-up information” (2 members of the public [79]). “I’d like to be notified anytime anybody accesses my medical records. Even if it’s my primary care physician... I’d either be notified through email or whenever you log on... When you log on, you should be able to see a list of everybody who’s accessed your file” (patient [74]). “What does the quantity look like? I mean, if we are getting 10 emails a day, we might get annoyed” (member of the public [11]).
Associated support [11,19,32, 36,40,41,43, 45-48,50-53,56, 59,68,69,73,74, 76,78,79,81-83, 85,86,88,90,92, 95,100,103,105, 108,114,123,127, 129,132,133,138, 139,141,143,150-153, 155,158,160,165, 171,172,174-183]	Support that is electronic [73,74,76,78,81,88,143,180] Support that is delivered by mail [32,59,78,158] Support that is face-to-face [43,59,79,83,139,160,175,177] Support that is delivered through technology [48,52,152] Support that is delivered traditionally [41,43,47,59,105] Support that is delivered through social media [88,108,132,172] Support that is delivered through emails [138,158,171] Support that is delivered through newsletters [69,83,171] Support that is delivered through academic and health institutions [40,85,108,132,141] Support that is delivered through health [11,19,32,40,45,50,56,59, 68,69,76,82,83,100,123,143,150-153, 155,165,174,176-178,183] or research [123,177] professionals and peers [51,76,92,129,132,143,153] Examples of technological supports include the following: Patient portals [114,123,158,172,175] Public databases [53,158] Websites [41,53,69,83,88,90,127,138,141,181] Web-based FAQ^a^ section [88,103] Short videos [11,36,103,132,174,179] Mobile apps [73,86] Examples of physical support included the following: Posters [36,95,123,176,181] Flyers [46,53,59,132,133,176]	“They [care providers] all use very strange words and it’s in one ear and out the other, and then when you get home, you have forgotten. But now, you can check again and you can look it up on the internet” (patient in conditions of vulnerability [175]). “I like how it’s (the electronic version) broken up so it’s easier to read. It’s less intimidating upon first glance than a packet of paper” (patient [73]). “If someone is telling face-to-face, it’s easier to motivate or convince the person. But if it’s some odd papers, sometimes you just skip the part that you didn’t need” (member of the public [79]). “Having a website is cost-effective because if people are interested they can go on it and have a look; if they're not then they don’t have to. Leaflets and things like that, I think, are expensive and unnecessary because 95 per cent of them will just end up at the bottom of a bird cage” (member of the public [105]). “Well then you’d feel that you were doing something that was very worthwhile wouldn’t you, you’d think you were part of it, instead of just wondering what’s going to happen, even a website we could come on and just see what’s happening, to keep us updated” (biobank participant [83]). “[I]f there’s sort of a portal to a web-based feedback that’s easy for physicians to use in the little time they have during the day, that would be good” (oncologist familiar with rapid learning systems [123]). “For me, it [consent portal]’s a must because it’s kind of a control thing. I would be able to see who’s using it and why” (member of the public [11]). “And if it works for me, I can point [to] my other nephews and cousins and everybody else and say: ‘Go try this out. Go see these people.’ Because I want to be a spokesman and I will say, you know, ‘This is what works. This is how I combat this or that.’ And I’d have an avenue to say, ‘Hey, go try that program out’” (patient from an Indigenous community [92]). “[...] To perceive leaflets as light reading while awaiting their appointments: ‘People pick them up and read them while they are waiting and them put them back’” (patient [59]). “The problem I have is that not everybody has a cell phone. Not everybody has access to electronics, and probably the people who are most underserved are those people. Probably the socioeconomic group odds are they don’t have money to buy these fun things, or they don’t have the education to be able to use them. So they’re left in the dark, and they’re probably the ones that are most easily taken advantage of” (oncology patient [114]).
Format [33,55, 56,65,72,73,91, 103,116,127,160, 167,174-176,184,185]	Brief [127,160,167,174,176] Interpreted [55,127,175,176,184,185] Layered to permit little or more information [33,56,73,91,103,127]	“If someone can answer, ‘Here’s where it’s stored, here’s how we use it’ in simple ways, not this 30-page agreement. Very simply [...]” (early adopter of eHealth technology [167]). “I sort of wish that I could provide some second bulleted point of one page that was like, ‘In plain language this is what you've just agreed to’” (researcher [127]). “The average person might not understand the meaning of all of [the results] so [results] would have to be returned in a format they can relate to” (researcher [127]).
Wording [11,31, 32,39,44,46,51, 54,56,63,65,70, 72,73,76,79,81, 87,92,93,99-101, 103,105,111,114, 127,130-132,134, 139,143,160,167, 172,173,175,176, 186-188]	In accessible, plain language [44,46,56,65,72,73,76,79,81, 87,92,93,99,100,103,105,111, 114,127,130,132,134,139,160,167, 173,175,176,188] Attention to levels of literacy [11,32,56,131,172,176,187] Use of mother tongue [51,70,92,188] Attention to special needs [101,143,176] Attention to cultural competency [51,92,131,186] Attention to tone [54,103] With explicit language [39,65,81,103,187], use of visual supports [103,176] and examples [103,176] Impartial and uncensored [31]	“Researchers should state exactly what is being done with data and make it simple for people to understand” (citizen council member [93]). “This [Health Care Information Directive] is too busy, it’s too much. If I’m sick, I friggin’ don’t want to be bothered with it... Look at this. English is my first language. How would somebody whose mother tongue is something other than English? [sic] It’s too complicated” (senior citizen [100]). “In terms of getting the information out to Alaskan Native people, just providing this in a very clear manner about what it is, what it means, what it can do for our system, what it can do for them individually. So, I think that, again, transparency is really huge” (health care provider [92]).

^a^FAQ: frequently answered questions.

#### Communication Approaches That Are Ongoing and Varied

To raise awareness and education, continuous communication methods (Table 4, row 1) as well as citizen forums were deemed appropriate strategies by all stakeholders, including patients and members of the public, as the best ways to reach all citizens “[...] before they actually become patients” [42]. For generic content for the purpose of informing with regard to a particular data set or data use or for specific content, mixed strategies with recourse to traditional media, social media, websites, patient portals, phone lines, posters, and flyers were proposed, again with the intention of reaching as many individuals as possible (Table 4, row 2). There was a concern that certain groups of citizens may be excluded if web-based communication is the only approach used [73,105,114]. Others favored information on demand as a cost-effective way for individuals to obtain the information needed [105]. Some underlined the virtue of centralizing the information to avoid being overwhelmed by the amount of information on the web [33,105,129]. Bernaerdt et al [175] outlined obstacles to the use of e-portals by “susceptible patients” and proposed recommendations for their design, an initial face-to-face encounter, and ongoing education.

#### Frequency

There was no consensus on the appropriate frequency, but the concept of continuous or periodic communication was frequently raised, with some practical suggestions to link to other communications and health encounters (Table 4, row 1). Overby et al [41] found that 51% of respondents stated that regular updates increased their likelihood of participating in a biobank [41].

#### Associated Support

Many stakeholders emphasized providing support around the communications (Table 4, row 2). Examples of institutional and technological support were proposed but the need to be able to converse directly with a person, whether a peer, a health professional, or a research coordinator, was deemed important. Overall, stakeholders, including the European Commission [203], encouraged person-centered approaches to communications [51,74]. Rake et al [179] presented a model of personalized consent flow *as a starting point to meet all requirements for sharing personally collected and controlled health data for research* [179]. In the context of biobanking, Dirks et al [78] emphasized the importance of not making assumptions regarding preferences about communication but to “[…] instead consider using communication strategies that use iterative inquiry to learn about and engage communities in which they [researchers] wish to conduct research.”

#### Format

Ideal formats of communications were deemed to be brief, accessible to all, notwithstanding impairments such as visual handicaps, and included an interpretation of results (when applicable) (Table 4, row 3). Of note, 43% of blind or low-vision respondents to a national survey exploring the views of persons with disabilities about participation and barriers to participation in precision medicine research agreed with the statement, “information about medical research is not accessible to me” [101].

In the context of eHealth, it was suggested that we need to move away from traditional terms and conditions that are infrequently read by users. The latter were reported to not be written in a way to engage people as it often does not use basic language or sufficient concise content, but at the same time, not explicit enough on certain aspects considered essential by the participants [65,167]. An interesting example involving layered communication was proposed in the context of communicating with patients about software to enhance privacy in secondary database research involving record linkages [103]. This strategy, which uses expandable text and on-demand definitions, allows to provide information to those who want it while reducing on-screen text for those who feel overwhelmed [103].

#### Wording

Stakeholders suggested considerable advice on the question of wording (Table 4, row 4). Nelson et al [176] proposed a whole framework to communicate health data that emphasizes the importance of plain language devoid of medical jargon and provide contextual information to assist interpretation. Communications should be explicit and consider special needs, native language, cultural competencies, tone, and level of literacy. Some have suggested dedicated resources to a phone line for direct access to information and explanations [53]. Patients [103] and other stakeholders [176] proposed the use of visual aids and inclusion of examples. Of interest, we came across a single stakeholder (member of a citizen jury) who raised concerns about the choice of certain terms and their legal implications [97].

### When to Communicate: Situations Where Communication Becomes Necessary

#### Overview

When discussing the secondary use of health data, stakeholders often referred to situations in which they considered communication necessary (Table 5). The 4 categories of situations that could or should trigger a communication, reaffirm themes that emerged around the types of information desired (Tables 2-3) and foreshadow, to a certain extent, the ideal format (Table 4).

**Table 5:** Situations where communication becomes necessary: description and examples of stakeholders’ perspectives.

Situations	Description	Exemplary citations of different stakeholders extracted from reviewed studies
Before the reuse of data [40,53,59, 67,74,80,81,86, 91,97,108,138, 144,145,152,158, 166,167,189-192]	To exert control on the secondary use of one’s data [40,67,81,91,152,189,190,192] When the reuse involves the private sector [53,81,97,158,191] When a minor participant reaches the age of majority [67,189] When the data are sensitive [53,59,74] In the context of public health emergencies when consent is not required [108]	“The most appropriate way is to inform the patient every time their data moves to the researcher or moves for a purpose and give them a chance to opt out or opt in each time. It may not be the most ideal for the company, but it’s much more ideal for the patient” (expert opinion regarding unregulated mobile app research [81]). “I don’t like it [one-time broad consent]. That’s just me because I mean it’s just like you sign the form once and you never see it again and then later on in life it ends up biting you in the ass cause well you signed the form once and you never saw it again. But someone goes out and dusts off your records and says, ‘Hey look here.’ I’m like, ‘Well Goddam I guess I did sign it.’ And you can’t do anything about it. There’s no option” (patient [40]). “I’m down with that... People can do whatever they want with our data... But what you’re trying to tell me is you’re now doing research that will put my name back on the data I gave you. In a way, you’re not just doing research on my data. You’re doing research on my data that will add data to my data that I didn’t give you for a reason” (early adopter of health technology [167]). “In an ideal world I would include that a company, when they share and sell the data, would need to have a site that users could access to see with whom their data has been shared” (patient advocate regarding unregulated mobile app research [81]).
When individual results become available [51,54, 56,69,81,83,91, 98,144,193]	Results that are actionable [81,83,91,144] or not [56] Incidental findings with clinical relevance [54,193] or not [193]	“Yes, definitely. The reason that this [cardiac arrest] doesn’t bother me anymore in daily life is that the blood clot was taken out and they explained to me what had happened. What had gone wrong in my body. I could see it clearly on the monitor during the catheterisation. So you finally know what it was that made you feel unwell. That was really nice. So in ninety percent of the cases I’d say, ‘tell me everything you can find about me, please’” (patient survivor of cardiac arrest [54]). “Well, I was pleased with it and I’m a bit like, that’s one of the incentives for me to go in for I was interested to know how well I was and I was also interested to know about my cholesterol as well because my father had really, really high cholesterol and I’ve never had mine done, so I thought, ‘well, that’s a way to find out what mine is’” (biobank participant [83]). “I would have liked to have had a more detailed summary than we actually got. I think there were other things that they could have given and, for example, had there been any major medical problems I think it would have been good if they’d have pointed those out at some stage or other” (biobank participant [83]). “It may not be a guarantee that this will happen, but one of the key issues in a disease such as this... is early identification and spurring people to action. Melanoma is probably one of the cancers that kills a lot of people, I would imagine because they aren’t aware of it and don’t act early enough... So, if he has an algorithm that’s more than 50% accurate, it’s imperative that he let the individuals be aware” (patient advocate regarding unregulated mobile app research [81]). “If that gentleman thinks he’s the carrier for something and he’s not, he needs to know that” (genetics specialist on the subject of false paternity [193]).
When new global research results become available [76,78,98,129, 144,156,158,186]	To maintain trust and community engagement [144,186] Research conclusions may influence a person [98,129] or a community’s actions [76,186]	“[...] A lot of the researchers were always promising verbally that they were going to share the information with you and... more than half the time they never see that the results of the data after they leave your community. That’s part of the reason why a lot of the Natives in small communities don’t trust the researchers” (member of an Indigenous community [144]).
In the occurrence of a breach in confidentiality [52,73,97,164]	N/A^a^	“If my health information is compromised, how will I be informed?” (patient [73]). “If you do the wrong thing, you should face the consequences... then maybe they won’t do it again” (citizen jury [97]). “Certainly, there is reportability back to the Institutional Review Boards [IRB]. There is probably reportability back to our audit committee of our board, which oversees compliance in Health Insurance Portability and Accountability Act [HIPAA] at the very minimum, depending if it’s identified... I mean, it could go all the way out to notification...Certainly notification to the patient if it's identified, but also perhaps the government agencies” (institution compliance officer [52]).

^a^N/A: not applicable.

#### Before the Reuse of Data

In the context of data sharing, many patients (67%) believed that they should be informed before their data were reused [86,192], and this feeling was enhanced when the data were viewed as sensitive [53,74] (Table 5, row 1). Sharing data with the private sector raised concerns [119,194]. In the context of Canadian citizens participating in a week-long dialogue, “[...] 56% of participants indicated that their tissue samples should never be used or that they must always be asked if profit is involved” [53]. Another interesting situation is that of minor participants who may want to be informed of past, current, and future data uses at the time of their majority [67,189].

#### When Individual Results Become Available

The return of individual results and the management of clinically important incidental findings have been largely discussed in the context of biobanking, but appear important in other contexts, including administrative databases [54,97], digital data [153], and mobile apps [81]. Some stakeholders highlighted how these communications—whether generic when global research results become available or specific when new individual research results become available— were deemed important to them as they would permit them to act on their own health or the health of their communities (Table 5, row 2). Finally, a few stakeholders deemed it necessary to communicate in the case of a breach of confidentiality.

## Discussion

### Principal Findings

**Figure 2:**
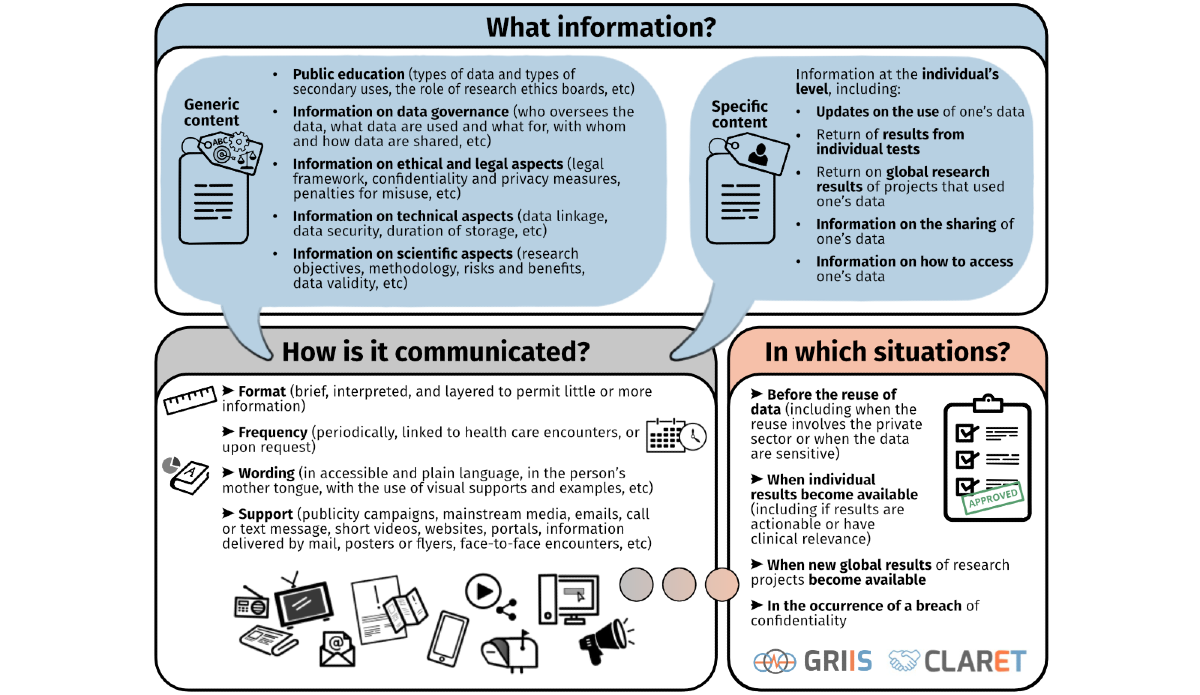
Summary of the scoping review’s findings.

This scoping review presents an extended analysis of the literature on patients, public, and other key stakeholders’ perspectives on transparency and secondary use of health data. It reports findings from 178 studies with various designs published over the past 2 decades. This extensive work is the first to map the views of a broad range of stakeholders on what information should be communicated to data contributors and how and in what situations to ensure transparency with the secondary use of health data. Figure 2 summarizes the findings of this study. There was a major overlap in the perspectives of different stakeholders. Some groups expressed opinions that reflected specific needs, such as data sovereignty in the context of Indigenous community data or access to results for patients with rare disorders. Overall, communication was deemed crucial for many purposes, including educating patients and members of the public on potential benefits, giving some control over data use as a form of reciprocity and as a condition to build and maintain trust. Elements that should be communicated include generic content, such as governance and regulatory frameworks, scientific aims, potential future uses of the data, and specific content that is relevant to each person with regard to the use of their data. Regarding methods of communication, broad approaches were generally favored, such as nationwide publicity campaigns, mainstream and social media for generic content, and mixed approaches for specific content, including websites, patient portals, and face-to-face encounters. Patients and members of the public rarely specified on whom the onus for these communications would fall, but we can imagine a shared responsibility according to intent and means between governmental, national, health care organizations, other large data custodians, and, to a lesser extent, individual researchers. Content should be customized to the individual as much as possible with regard to the length, avoidance of technical terms, cultural competence, and level of detail.

### Interpretation of Findings

At this moment, in Western societies, sharing both generic and specific content seems necessary to achieve the goals of raising awareness, creating and maintaining trust by respecting the *moral concerns of donors* [110], and addressing the specific information needs of populations that have experienced racism and research violations [166,186,190]. Raised awareness and education are fundamental for members of the public to understand the legitimacy of various secondary uses of health data [58,79,134,163,194,195]. However, the data suggest that the current knowledge deficit is important [196,197]. In the international survey by Milne et al [85], providing transparent information about who will benefit from access to genomic data was the most important measure for increasing trust. There is an ongoing perceived need for control over the secondary use of health data and an aspiration for *greater equity in the science-public relationship* [31]. These findings confirm the conceptual framework by Brall et al [147] of individual willingness to participate in personalized health research and emphasize certain elements. The framework identified 20 elements pertaining to attitude, motivation, utility of results, data sharing and use, data management, and data governance that can have an impact on individual willingness. All these elements are presented in our review. Their relative importance could evolve with society’s greater understanding of data, measures taken to make their reuse secure, and confirmation of the benefits of secondary use. Achieving the objective of transparency needs to address its various facets whether it is “informational transparency” defined by Aitken et al [31] as “requiring disclosure of information on which decisions are based,” “participatory transparency” defined as “enabling public participation in decision-making processes,” and “accountability transparency” defined as “decision-makers are held accountable.” Any step toward greater communication and transparency should be carefully considered to ensure relevance and quality and decrease the risk of negative backlash [204].

A narrative review of the literature highlights the fact that social licenses for data-intensive health research cannot be assumed [205]. Trust and its absence had a considerable impact on the acceptance of the secondary use of health data without specific consent [136,191,198]. Muller et al [206] emphasized the merits of using the concept of social license as a reference for ethical governance and highlighted the strategic importance of dialogue with patients and members of the public. Research is ongoing to define what secondary uses of health data are within a social license and how perspectives could change with greater transparency [207]. On the basis of 8 focus groups with Canadian members of the public, Paprica et al [194] concluded that “[r]esearchers and organizations that hold health data should engage with members of the public to ensure that research is aligned with social license, particularly where there is private-sector involvement.” These findings are also reflected in a similar study of deliberative focus groups conducted with United Kingdom citizens [208]. Furthermore, we would do well to not assume and rather gather more evidence to support the idea that transparency and trust will necessarily go hand in hand [20].

Reciprocity was also a transversal theme in this review and is likely a foundation on which to build this common understanding with different communities and populations. Indeed, Sexton et al [34] linked trust and reciprocity and raised the concern that “[f]aceless processes of governance are increasingly foregrounded over traditional relational bonds.” Hobbs et al [83] explored the privacy-reciprocity connection, whereby participants attached certain conditions to their research donations. Patient engagement is another form of reciprocity that could increase trust [31]. Finally, reciprocity from the private sector appeared to be an important condition for sharing health data [136,191].

The return of individual results, particularly if perceived as potentially actionable, was a commonly cited example of a specific communication that patients and members of the public wanted, and this was substantiated by other research [209,210]. For some but not all, it was a motivation to donate health data, including genomic data [199]. What is less clear is whether, in the absence of the return of individual results, the need for information could be satisfied by communication on aggregated or global research results.

We also tried to distinguish whether individuals wished to receive specific communication about the secondary use of information by a single research project or by a group of projects with similar features. However, that distinction was not clearly present in our analysis. It could be interesting to confirm that an intermediate level of information is linked to the category of projects (eg, academic research, research with genomic data, and research with a private partner) that may or is using the data could meet individuals’ need for information without excessive use of resources [192]. This would also align well with a metaconsent model, a promising dynamic approach to information and consent in the context of LHS [11].

There will be no one size fits all—some individuals wanted regular communications by varied means and at specific times, for example, each time their data were used, whereas others wished for basic information to be available on demand so that resources can be allocated elsewhere. Individual factors, including age, ethnicity, gender, education, general and scientific culture, curiosity, and motivations to participate in research, were key to explaining the variety of views on the subject [56,59,83,151,158,166]. Biobank participants, for example, may have a more altruistic outlook and a reduced need for the return of information than members of the public at large who are less aware of the risks and benefits of research. It will also be important to pay attention to the “unheard voices” of people who are marginalized in society [169]. Public deliberation is an interesting approach to better understand specific population needs and preferences for notifications about data sharing [114]. Any communication strategy will need to be patient-centered and modifiable so that low information seekers are not overwhelmed by unwanted communication, and high information seekers can equally meet their needs [11,69]. Communications should also be adapted to the needs of people with disabilities, especially considering that technology is creating an information accessibility gap as it tends to replace face-to-face communication [101]. Interesting models of communication include the frequently asked questions section that provides layered information so that an individual could obtain as much or as little information as desired [103] and the OPT-IN framework by Nelson et al [176] “[...] to help guide communicators with selecting and presenting data to lay audiences, taking into account the broader communication perspective.” Guidelines on how to discuss the protection and use of personal health information would also be welcome [59].

### Findings in Relation to Broader Context

There are other noteworthy initiatives to help us pave the way forward and find a balance between information needs, trust, and societal benefits from the secondary use of health data [200]. We would like to highlight 4 key features: patient or public engagement, harmonization of policies, the importance of user-centered informational tools, and innovations in data security.

First, transparent governance and patient engagement are increasingly being proposed as important avenues toward social license. Paprica et al [87] proposed the minimal specifications for data trust. Participatory governance is a key concept, as exemplified in the Academic Research Network of Diabetes Action Canada [72], the Research Data Alliance COVID-19 Working Group guidelines [201], and in 1 of the 7 recommendations from the work by Courbier et al [76] with patients with rare diseases. Participatory governance is also necessary to respect the Principles for Indigenous Data governance [201].

Second, the adoption of coherent policies and practices can help harmonize different initiatives and reduce risks, such as privacy breaches [44]. National or institutional standards such as Quebec’s Fonds de Recherche Standards for Data Access Centers [211], New Zealand’s integrated data infrastructure [166], and the United Kingdom’s data registry standard [212] are a few examples of initiatives that can serve as models.

Third, educational tools will be required to raise awareness among all stakeholders, with a focus on members of the public and tailored to specific populations. Such large-scale initiatives could address the frequently expressed need for information to help individuals control their use of data. Indeed, studies have demonstrated that once informed of regulatory safeguards, presented with conditions around use [6], informed of impacts [213], or sensitized to logistic hurdles, opinions regarding individual consent and control can shift.

Fourth, we should build on existing communication strategies. The Health Care Information Directive is an example of a patient decision aid that aims to delineate the level of health information that an individual is willing to share [100,214]. Caine et al [74] derived 6 implications for the design of a patient-centered tool to allow individual choices in the disclosure of health data from patient interviews. In their systematic review of the design of patient aids, Vaisson et al [215] proposed a user-centered design framework that could help design future aids to track the secondary use of one’s health data. The proposed concept interface enables contextual control by allowing patients to set their privacy levels in the context of viewing events within an electronic health record. Fagerlin et al [216] proposed 10 pragmatic recommendations to help patients make decisions when faced with complex information on risks and benefits that could be applied to making decisions about data sharing. On a more theoretical level, Zikmund-Fisher [217] proposed a standard taxonomy of risk concepts that could inform or evaluate future communications. Riso et al [70] outlined a framework with 6 core values to improve the ethical standards of data-sharing platforms. For example, the GA Registry [218] is patient-led and permits large-scale data sharing while letting users decide which data sets to share and with whom. Exemplary projects and patient portals include the tailored, ongoing communication of the CHRIS longitudinal study [202], NIH-funded *All of Us* Research Program [219], *Connect Care* (Canada) [220] that allows bidirectional interaction with the patient, *Sundhed* (Denmark) [221], and *Health data hub* (France) [222].

Finally, ongoing innovations in data security will be important to enhance privacy and trust in all these initiatives. Examples include CrowdMed, a blockchain approach proposed by Shah et al [180] and MiNDFIRL, a software to enhance privacy in secondary database studies [103].

### Limitations

Our search yielded most articles originating from North America and Europe and documenting Western contexts and attitudes; therefore, our review may not entirely represent other societies. In addition, although we cited research conducted with stakeholders from Indigenous communities, we cannot conclude that the views collected are representative of all Indigenous communities. Furthermore, many earlier articles focused on research conducted in the context of biobanking. We restricted our analysis to articles that explored stakeholder perspectives with a focus on the future use of samples or data. During thematic analysis, we were able to conclude that many of the perspectives overlapped with research conducted in other contexts, such as administrative databases, data mining, and mobile app. This suggests that for the person concerned by the secondary use of health data, there are similarities between the research contexts of biobanking and newer contexts.

### Conclusions

This scoping review highlights the diversity and extent of patient and public expectations regarding information on the secondary use of health data. It also attests how and in which situations the communication of different types of information could contribute to the objective of greater transparency. These findings suggest that governing bodies should actively invest in widespread and targeted activities to increase public awareness and understanding about the secondary use of health data. Modern societies would do well to foster a culture of information and health literacy, which in turn is necessary for individual empowerment in the context of health care. Indeed, our results highlight the importance of patient, citizen, and community engagement in different secondary uses of health data [104]. This is particularly true for some populations such as Indigenous communities that have developed their own models of data governance [223].

Future studies should consider building on existing models to develop thoughtful strategies for communicating with the secondary use of health data. This review did not identify resources specifically considering the LHS context; therefore, the impact of such a framework on the desires of patients and members of the public remains to be explored. To support the implementation of LHSs in Quebec (through a transparency portal), the next steps would be to validate some of the results with patients and members of the public, including the following: (1) the need for both types of content (generic and specific); (2) the level of generic content to meet information needs and foster and sustain trust; and (3) the resulting most appropriate format considering resource use and development of web portals to support LHSs [224]. Other research could also validate how we can match actual citizen and patient needs with congruent types of knowledge and informational tools (Table 2 in the study by Zikmund-Fisher [217]), and how the personalization of communications meets the demands of different regulatory bodies, for example, the recommendations of the European Commission [203]. Another important question is whether transparency leads to unexpected outcomes, such as desensitization and “systematic exploitation” [111]. Finally, these strategies should be evaluated to ensure that they reach their objectives, notably with regard to transparency and even improving health outcomes at the individual and system levels. Beyond just informing members of the public on the secondary use of their data, we should aim to foster a culture in which all citizens are given tools to participate in their health care and determine research priorities [139].

## Appendix

Multimedia Appendix 1 PRISMA-ScR Checklist.

Multimedia Appendix 2 Research strategies.

Multimedia Appendix 3 Characteristics of the articles included in the scoping review.

## Abbreviations

LHS: learning health system
PRISMA-ScR: Preferred Reporting Items for Systematic Reviews and Meta-Analyses extension for Scoping Reviews

## Data Availability

The data generated and analyzed during this study are available from the corresponding author upon reasonable request.
