# Supplementary material for: Patients’ and Members of the Public’s Wishes Regarding Transparency in the Context of Secondary Use of Health Data: Scoping Review
Source: J Med Internet Res. 2023 Apr 13;25:e45002. doi: 10.2196/45002 (PMC10141314; doi:10.2196/45002)
Supplement: Multimedia Appendix 2 [file jmir_v25i1e45002_app2.docx]

**Multimedia Appendix 2.** Research strategy

### *Medline, CINAHL, PsycINFO*

(patient* OR public* OR citizen*) AND ("health data" OR "healthcare data" OR "health care data" OR "medical data" OR "data sharing" OR "data-sharing" OR "data access" OR "data transfer" OR "data use*" OR "data reuse*" OR "data utilization" OR "data utilisation") AND (attitude* OR view* OR perspective* OR opinion* OR position*) AND (inform* OR transparen* OR communicat* OR disseminat* OR aware* OR notif* OR educat*) AND (research OR "secondary use" OR "secondary utilisation" OR "secondary utilization" OR "learning health system*" OR "learning healthcare system*" OR "learning health care system*" OR "learning healthcare project*" OR "learning health care project*" OR "learning health" OR "learning healthcare" OR "learning health care" OR "big data healthcare")

*Note: No additional restriction applied.

### *Scopus, Cochrane Library*

TITLE-ABS-KEY ((patient* OR public* OR citizen*) AND ("health data" OR "healthcare data" OR "health care data" OR "medical data" OR "data sharing" OR "data-sharing" OR "data access" OR "data transfer" OR "data use*" OR "data reuse*" OR "data utilization" OR "data utilisation") AND (attitude* OR view* OR perspective* OR opinion* OR position*) AND (inform* OR transparen* OR communicat* OR disseminat* OR aware* OR notif* OR educat*) AND (research OR "secondary use" OR "secondary utilisation" OR "secondary utilization" OR "learning health system*" OR "learning healthcare system*" OR "learning health care system*" OR "learning healthcare project*" OR "learning health care project*" OR "learning health" OR "learning healthcare" OR "learning health care" OR "big data healthcare"))

*Note: No additional restriction applied.

### *PubMed*

(patient OR patients OR public OR publics OR citizen OR citizens) AND ("health data" OR "healthcare data" OR "health care data" OR "medical data" OR "data sharing" OR "data-sharing" OR "data access" OR "data transfer" OR "data use" OR "data uses" OR "data reuse" OR "data reuses" OR "data utilization" OR "data utilisation") AND (attitude OR attitudes OR view OR views OR perspective OR perspectives OR opinion OR opinions OR position OR positions) AND (information OR informed OR transparency OR transparent OR communication OR communicated OR dissemination OR disseminated OR awareness OR aware OR notification OR notified OR notify OR education OR educated) AND (research OR "secondary use" OR "secondary uses" OR "secondary utilisation" OR "secondary utilization" OR "learning health system" OR "learning health systems" OR "learning healthcare system" OR "learning healthcare systems" OR "learning health care system" OR "learning health care systems" OR "learning healthcare project" OR "learning healthcare projects" OR "learning health care project" OR "learning health care projects" OR "learning health" OR "learning healthcare" OR "learning health care" OR "big data healthcare")

*Note: Restriction to publications in the three two years only to capture most recent indexations.
