# Supplementary material for: Patients’ and Members of the Public’s Wishes Regarding Transparency in the Context of Secondary Use of Health Data: Scoping Review
Source: J Med Internet Res. 2023 Apr 13;25:e45002. doi: 10.2196/45002 (PMC10141314; doi:10.2196/45002)
Supplement: Multimedia Appendix 3 [file jmir_v25i1e45002_app3.docx]

**Multimedia Appendix 3.** Characteristics of the articles included in the scoping review (n=178)

| Articles | Year of publication | Country where study was conducted | Study design | Perspectives reported in the study |
| --- | --- | --- | --- | --- |
| Cumyn et al [6] | 2021 | Canada | survey | public |
| Kim et al [8] | 2015 | United States | survey | public |
| Cumyn et al [11] | 2021 | Canada | qualitative study | public |
| Jagsi et al [18] | 2017 | United States | survey | cancer patients |
| Kisekka et al [19] | 2021 | United States | survey | patients |
| Carr and Littler [30] | 2015 | United Kingdom | commentary | authors |
| Aitken et al [31] | 2016 | United Kingdom | qualitative study | public; stakeholders |
| Goytia et al [32] | 2018 | United States | qualitative study | patients; cliniciens |
| Jones et al [33] | 2020 | United Kingdom | data governance standards; recommandations | authors |
| Sexton et al [34] | 2017 | United Kingdom | qualitative study | university data managers; academic researchers; research board members; students |
| Terry et al [35] | 2014 | Canada | qualitative study | cliniciens/healthcare practitioners |
| Hepgul et al [36] | 2019 | United Kingdom | response letter | authors |
| O’Brien et al [37] | 2019 | United States | survey | patients |
| Jao et al [38] | 2015 | Kenya | qualitative study | research stakeholders |
| Lemke et al [39] | 2010 | United States | qualitative study | public; biobank participants |
| Mamo et al [40] | 2013 | United States | qualitative study | patients |
| Overby et al [41] | 2015 | United States | survey | patients |
| Richter et al [42] | 2021 | The Netherlands; Germany | survey | patients; public |
| Soni et al [43] | 2020 | United States | qualitative study | patients |
| Watson et al [44] | 2020 | United States | ethical opinion paper | authors |
| Wetzels et al [45] | 2018 | The Netherlands | qualitative study | patients |
| Ipsos MORI [46] | 2007 | United Kingdom | mixed-method study | public |
| NICE Citizens Council [47] | 2004 | United Kingdom | qualitative study | public |
| Westin et al [48] | 2008 | United States | survey | public |
| Atkin et al [49] | 2021 | United Kingdom | qualitative study | patients; public; carers; healthcare staff |
| Darquy et al [50] | 2016 | France | mixed-method study | rare diseases patients;  family members |
| Tindana et al [51] | 2020 | Ghana; Uganda; Zambia | qualitative study | biobank participants |
| Manion et al [52] | 2009 | United States | qualitative study | regulatory stakeholders |
| Saxena et al [53] | 2006 | Canada | qualitative study | public |
| Bak et al [54] | 2021 | The Netherlands | qualitative study | survivors of sudden cardiac arrest; next-of-kin |
| Cheung et al [55] | 2020 | United States | mixed-method study | scientific and legal experts |
| Hishiyama et al [56] | 2019 | Japan | survey | public |
| Garrison et al [57] | 2019 | United States | qualitative study | indigenous communities |
| Jao et al [58] | 2015 | Kenya | qualitative study | research stakeholders |
| Shickle et al [59] | 2002 | United Kingdom | mixed-method study | public; patients |
| Adanijo et al [60] | 2021 | United Kingdom | qualitative study | mental health patients |
| Richter et al [61] | 2021 | Germany | survey | patients |
| Demotes-Mainard et al [62] | 2019 | France | qualitative study; recommandations | stakeholders |
| Haga et al [63] | 2011 | United States | qualitative study | public |
| Joly et al [64] | 2015 | Canada | survey | public |
| Jones et al [65] | 2019 | United Kingdom | qualitative study | public |
| Jones et al [66] | 2019 | United Kingdom | ethical framework | authors |
| McCormack et al [67] | 2016 | 16 countries (as part of the EURORDIS network) | qualitative study | rare diseases patients |
| Nair et al [68] | 2004 | Canada | qualitative study | patients |
| Meulenkamp et al [69] | 2010 | The Netherlands | survey | public; patients |
| Riso et al [70] | 2017 | European countries | ethical framework | NA |
| Sanderson et al [71] | 2017 | United States | survey | public |
| Willison et al [72] | 2019 | Canada | governance framework; policies | authors |
| Harle et al [73] | 2018 | United States | qualitative study | patients |
| Caine et al [74] | 2015 | United States | qualitative study | patients |
| Clarke et al [75] | 2021 | United Kingdom | survey | public |
| Courbier et al [76] | 2019 | Multiple European countries (as part of the EURORDIS network) | survey | rare diseases patients; family members |
| Damschroder et al [77] | 2007 | United States | mixed-method study | patients |
| Dirks et al [78] | 2019 | United States | qualitative study | native community |
| Drobotowicz et al [79] | 2021 | Finland | qualitative study | public |
| Franklin et al [80] | 2020 | United States | survey | cancer patients |
| Hammack-Aviran et al [81] | 2020 | United States | qualitative study | patients/research advocates; researchers; regulatory professionals; mobile app/device developpers |
| Hassan et al [82] | 2020 | United Kingdom | qualitative study | public |
| Hobbs et al [83] | 2012 | United Kingdom | qualitative study | biobank participants |
| Kim et al [84] | 2019 | United States | survey | patients |
| Milne et al [85] | 2021 | 22 countries | survey | public |
| Mursaleen et al [86] | 2017 | United Kingdom | survey | parkinson's disease patients |
| Paprica et al [87] | 2020 | Canada | essential requirements; data governance | people representing organizations and data infrastructure initiatives |
| Platt et al [88] | 2014 | United States | survey | public |
| Tauali i et al [89] | 2014 | United States | qualitative study | native hawaiian |
| Teng et al [90] | 2019 | Canada | qualitative study | public |
| Trinidad et al [91] | 2012 | United States | qualitative study | patients |
| Woodbury et al [92] | 2020 | United States | qualitative study | Alaska native tribal |
| NICE Citizens Council [93] | 2015 | United Kingdom | qualitative study | public |
| Lysaght et al [94] | 2020 | Singapore | qualitative study | public |
| Willison et al [95] | 2003 | Canada | mixed-method study | patients |
| Liyanage et al [96] | 2016 | United Kingdom | delphi study; data access framework | authors |
| Street et al [97] | 2021 | Australia | qualitative study | public |
| Barazzetti et al [98] | 2020 | Switzerland | qualitative study | patients; biobank recruiters |
| Rivas Velarde et al [99] | 2021 | Switzerland | qualitative study | public |
| Tracy et al [100] | 2004 | Canada | qualitative study | patients; public |
| Sabatello et al [101] | 2019 | United States | survey | blind/low-vision participants |
| Peppercorn et al [102] | 2020 | United States | survey | cancer patients |
| Schmit et al [103] | 2020 | United States | delphi study | patients |
| Tully et al [104] | 2019 | United Kingdom | qualitative study | public |
| Davidson et al [105] | 2013 | United Kingdom | qualitative study | public |
| Mazor et al [106] | 2017 | United States | qualitative study | patients; researchers; institutional review board; regulatory staff; research governance experts; healthcare system leaders |
| Cobban et al [107] | 2008 | Canada | ethical perspective | authors |
| McCradden et al [108] | 2020 | Canada | qualitative study | cancer patients; caregivers; providers |
| Kim et al [109] | 2014 | United States | data governance requirements | authors |
| De Vries et al [110] | 2019 | United States | qualitative study | public |
| Kasperbauer [111] | 2020 | United States | opinion paper | authors |
| Ipsos MORI [112] | 2016 | United Kingdom | qualitative study | public |
| Middleton et al [113] | 2019 | Canada; United Kingdom; United States | survey | public |
| Raj et al [114] | 2022 | United States | qualitative study | public |
| McCormick et al [115] | 2022 | United States | survey | patients |
| McCormick et al [116] | 2021 | United States | qualitative study | public |
| Richter et al [117] | 2019 | Germany | survey | patients |
| Geneviève et al [118] | 2021 | Switzerland | qualitative study | data sharing expert stakeholders |
| Kaufman et al [119] | 2009 | United States | survey | public |
| Mouton Dorey et al [120] | 2018 | Switzerland | qualitative study | physicians; research ethics committee members; sponsors; regulators; policy makers |
| Snell et al [121] | 2012 | Austria; Finland; Germany | qualitative study | public |
| Dimitropoulos et al [122] | 2011 | United States | survey | public |
| Mayo et al [123] | 2017 | United States | qualitative study | providers |
| Middleton et al [124] | 2020 | Canada; United Kingdom; United States | survey | public |
| Annas [125] | 2003 | United States | law policies | authors |
| Maiorana et al [126] | 2012 | United States | mixed-method study | patients; providers |
| McGuire et al [127] | 2012 | United States | qualitative study | research participants; research investigators; NIH employees |
| Colombo et al [128] | 2019 | Italia | mixed-method study | patients; public |
| Chung et al [129] | 2018 | United States | qualitative study | patients |
| Dove and Phillips [130] | 2015 | United Kingdom | legal frameworks | authors |
| Mbuthia et al [131] | 2019 | Kenya | qualitative study | health managers; providers; researchers |
| Douglas et al [132] | 2018 | United Kingdom | qualitative study | public |
| Alaqra et al [133] | 2021 | Sweden | qualitative study | eHealth experts stakeholders |
| Vezyridis et al [134] | 2019 | United Kingdom | qualitative study | health data researchers; general practioners; public |
| Muller et al [135] | 2022 | European countries | survey | patients; public |
| Shah et al [136] | 2021 | United Kingdom; Iceland; Sweden | qualitative study | public |
| Ormondroyd et al [137] | 2022 | United Kingdom | qualitative study | clinical, science, ethical, legal and public health stakeholders; ‘Direct-to-consumer’genetic tests providers; patients |
| Manhas et al [138] | 2015 | Canada | qualitative study | parents of pediatric patients |
| McCradden et al [139] | 2020 | Canada | qualitative study | public |
| Bromley et al [140] | 2020 | United States | qualitative study | research stakeholders |
| Tsai et al [141] | 2018 | Taiwan | expert opinion paper | authors |
| Page et al [142] | 2016 | Canada | survey | patients |
| Spencer et al [143] | 2016 | United Kingdom | qualitative study | patients |
| Hiratsuka et al [144] | 2012 | United States | qualitative study | native community |
| Bull et al [145] | 2015 | India; Vietnam; Thailand; South Africa; Kenya | qualitative study | stakeholders |
| Kaphingst et al [146] | 2006 | United States | qualitative study | breast cancer patients |
| Brall et al [147] | 2021 | Switzerland | survey | public |
| Kaufman et al [148] | 2009 | United States | survey | veterans patients |
| Kerath et al [149] | 2013 | United States | survey | patients |
| Master et al [150] | 2013 | Canada | survey | cancer patients |
| Ruiz-Canela et al [151] | 2011 | Spain; United States | survey | research participants |
| Tabor et al [152] | 2012 | United States | qualitative study | patients and families with Miller syndrome |
| Seltzer et al [153] | 2019 | United States | survey | patients |
| Eikemo et al [154] | 2022 | Norway | survey | patients |
| Burstein et al [155] | 2014 | United States | qualitative study | parents of pediatric patients; adult patients |
| Meulenkamp et al [156] | 2012 | The Netherlands | survey | researchers |
| Richards et al [157] | 2016 | United States | qualitative study | biobank participants |
| Tosoni et al [158] | 2021 | Canada | survey | patients |
| Rushmer et al [159] | 2011 | United Kingdom | qualitative study | patients; researchers; practice staff |
| Köngeter et al [160] | 2022 | Germany | survey | cancer patients |
| Clerkin et al [161] | 2013 | Ireland | qualitative study | patients |
| Tully et al [162] | 2018 | United Kingdom | qualitative study | public |
| Xafis [163] | 2015 | Australia | qualitative study | public |
| Hate et al [164] | 2015 | India | qualitative study | research participants; public health researchers |
| McGuire et al [165] | 2008 | United States | qualitative study | patients |
| Thabrew et al [166] | 2022 | New Zealand | qualitative study | patients |
| Cheung et al [167] | 2016 | United States | qualitative study | early adopters of emerging health technologies |
| Monaghan et al [168] | 2020 | Australia | qualitative study | general practitioners; practice nurses; practice managers |
| Mulrine et al [169] | 2021 | United Kingdom | qualitative study | public |
| Edwards et al [170] | 2011 | United States | survey | genetic researchers |
| Manhas et al [171] | 2018 | Canada | survey | parents of pediatric patients |
| Hivon et al [172] | 2017 | Canada | qualitative study | public |
| Jones et al [173] | 2020 | United Kingdom | data governance framework | authors |
| Riggs et al [174] | 2019 | United States | qualitative study | public |
| Bernaerdt et al [175] | 2021 | Belgium | qualitative study | vulnerable patients |
| Nelson et al [176] | 2011 | United States | consent framework; health policy | authors |
| Lucero et al [177] | 2015 | United States | qualitative study | minority community members |
| Piel et al [178] | 2018 | United Kingdom | commentary | authors |
| Rake et al [179] | 2017 | The Netherlands | viewpoint; consent model framework | authors |
| Shah et al [180] | 2019 | China | consent management framework | authors |
| Ballantyne et al [181] | 2019 | New Zealand | commentary | authors |
| Amr et al [182] | 2022 | Germany | survey | patients |
| Romano et al [183] | 2021 | Italy | survey | public |
| Mählmann et al [184] | 2017 | Switzerland | qualitative study | older adults |
| Neves et al [185] | 2019 | United Kingdom | qualitative study | healthcare professionals |
| James et al [186] | 2014 | United States | qualitative study | indigenous communities |
| Schraefel et al [187] | 2017 | United Kingdom | cover story | authors |
| Hripcsak et al [188] | 2014 | United States | recommandations | healthcare academics; policy makers; system stakeholders; patient groups |
| Jamal et al [189] | 2014 | United States | qualitative study | research participants |
| Jagsi et al [190] | 2019 | United States | mixed-method study | cancer patients |
| Braunack-Mayer et al [191] | 2021 | Australia | survey | public |
| Buhr et al [192] | 2022 | Germany | survey | chronic heart failure patients |
| Downing et al [193] | 2013 | United States | qualitative study | genetic specialists |
| Paprica et al [194] | 2019 | Canada | qualitative study | public |
| Amorim et al [195] | 2022 | Portugal | survey | rare disease patients; carers; healthcare professionals |
| Aggarwal et al [196] | 2021 | United Kingdom | survey | patients |
| Alrefaei et al [197] | 2022 | Saudi Arabia | survey | public |
| Belfrage et al [198] | 2022 | Sweden | survey | public |
| Milne et al [199] | 2022 | 22 countries | survey | public |
| Martani et al [200] | 2022 | Denmark | qualitative study | policy-makers; researchers; public administratives |
| Austin et al [201] | 2020 | Worldwide | recommandations; guidelines | authors |
| Mascalzoni et al [202] | 2022 | Italy | descriptive study | researchers |
